# Environmental Factors Associated with Altered Gut Microbiota in Children with Eczema: A Systematic Review

**DOI:** 10.3390/ijms17071147

**Published:** 2016-07-16

**Authors:** Carmen W. H. Chan, Rosa S. Wong, Patrick T. W. Law, Cho Lee Wong, Stephen K. W. Tsui, Winnie P. Y. Tang, Janet W. H. Sit

**Affiliations:** 1 Nethersole School of Nursing, The Chinese University of Hong Kong, Hong Kong, China; szemanwg@hotmail.com (R.S.W.); patricklaw@cuhk.edu.hk (P.T.W.L.); jojowong@cuhk.edu.hk (C.L.W.); winnie.tang@cuhk.edu.hk (W.P.Y.T.); janet.sit@cuhk.edu.hk (J.W.H.S.); 2 School of Biomedical Sciences, The Chinese University of Hong Kong, Hong Kong, China; kwtsui@cuhk.edi.hk

**Keywords:** childhood eczema, microbiome diversity, gut microbiota, allergy development, immune system, gene-environment interaction, environmental-host-microbial interaction

## Abstract

Eczema is a common skin condition that impairs children’s daily life activities and quality of life. Previous research shows that gut microbiome composition plays an important role in the development of eczema. The present review summarizes evidence on environmental factors related to altered gut microbiota in children with eczema. We searched Medline, PubMed, Embase, and the Cochrane database of Systematic Reviews through October 2015. The search strategy focused on articles published in peer-reviewed, English-language journals with no publication year limit. Only original studies and review articles that reported environmental factors on gut microbiome specific to eczema were included in this review. We selected six studies (total 1990 participants) for full review and identified that the composition of gut microbiota specific to eczema could be influenced by the following environmental factors: length of gestation, mode of delivery, type of feeding, method of treatment, number of older siblings, and other lifestyle factors. There has been inconsistent empirical evidence as to the modulatory effects of gut microbiota on immunological functions in children with eczema. Further research on the environmental-host-microbial interaction is needed to develop a strong base of knowledge for the development and implementation of prevention strategies and policies for eczema.

## 1. Introduction

Eczema, also known as atopic dermatitis, is a common childhood condition characterized by the inflammation of skin with intense itching [1]. It has been reported that children with eczematous skin lesions experience poor quality of life and encounter social difficulties at school [2]. They also have a greater risk of developing other allergic diseases, such as asthma in later childhood [3]. The prevalence of eczema in children is much higher in developed countries than in developing countries (up to 20% in Northern and Western Europe, Australia, and the United States vs. less than 2% in China) [4]. This higher prevalence in developed countries may be a result of gene-environment interactions and has led to increasing research interest and investigations into the risk and protective factors of eczema.

It is recognized that genetic factors, such as parental eczema history, make significant and important contributions to the occurrence of eczema in their offspring [5]. In addition, researchers suggest that environmental factors can act on the basis of genetic susceptibility to drive an increase in disease prevalence [6]. A study on the prevalence of allergic diseases among preschool children in Germany, following the country’s reunification, observed a stable disease incidence in West Germany but a steady rise in East Germany [7]. The authors attributed this rise to the adoption of a western lifestyle and environment in East Germany. Effective public health measures often lead to a decline of childhood infections in developed countries. However, according to the “hygiene hypothesis”, infectious agents include protective bacteria, the reduction of which may result in an increase of allergic and autoimmune diseases [8]. Epidemiological data of cross-sectional studies has shown evidence of the benefits of commensal bacteria to human body [9]. These findings highlight the important influences of early life exposures on the expression of childhood eczema.

Exposure to microorganisms is important in shaping the immune system, as the commensal bacteria in the gut are colonized through environmental exposures and play a pivotal role in defending the host from invading pathogens and other harmful agents. The human gut is a highly selective ecosystem colonized with different strains of bacteria, fungi, parasites, protozoa, and viruses along the intestinal tract [10]. Due to the advanced genomic sequencing technology, there has been a considerable body of empirical evidence showing a distinctive pattern of microbial diversity in eczematous children, with a higher count of aerobic bacteria, such as coliforms and *Staphylocccus aureus* and a decreased proportion of *Lactobacilli*, or anaerobes, such as *Bifidobacterium* or *Bacteroides* [11]. Changes in the microbial community may cause inhibitory immune responses to antigens, such as allergens. The establishment and maintenance of balanced intestinal microbiota can, therefore, facilitate the development of a strong immune system for basic physiological functions.

It is noteworthy that the infant gut colonization process involves bacteria from the mother and the surrounding environment [11]. The process may begin during gestation and continue over the first few years of life, which corresponds to a critical period of immune development and maturation. During these critical years, infants are exposed to numerous environmental factors, such as mode of delivery, type of feeding and sibship size, all of which have been suggested to influence the development and diversification of gut microbiota [12]. Since alterations of normal microbiota constituents may predispose infants to certain immune-related diseases later in life, the identification of environmental risk factors of low microbial diversity is an important first step for preventing eczema.

A few canine and mouse eczema models have been identified and validated, such as atopic Beagles and Nishiki-nezumi Cinnamon/Nagoya (NC/Nga) mice [13,14]. They are essential for the study of the mechanism and the development of eczema. However, studies on the interaction between gut microbiota and the development of eczema in the experimental models are limited and we cannot integrate the results with human data easily. Four review articles [15,16,17,18] on the development of allergic diseases such as eczema through the microbe effects on immune system were recently published. All were general literature reviews rather than systematic reviews. All of them did not provide a clear and comprehensive description of their search methods and inclusion criteria which created difficulty in assessing the scientific vigor of the evidence cited in their articles.

There is a need for a systematic and critical review of extant findings to guide clinical practice and future research in this area. To our knowledge, there is no review article focusing exclusively on the environmental risk factors of low microbial diversity observed in childhood eczema. This paper, therefore, aims at critically reviewing scientific articles that examine external factors contributing to altered gut microbiota in children with eczema.

## 2. Methodology of Review

### 2.1. Identification and Selection of Articles

A systematic review of the literature was performed in October 2015. The search strategy focused on articles published in peer-reviewed, English-language journals with no publication year limit. The databases used were Medline, PubMed, Embase, and the Cochrane Database of Systematic Review. Keywords used in each electronic database search included eczema and gut microbiota. Only original studies reported environmental factors on gut microbiome specific to eczema were included in this review. Editorial, case reports, dissertations, or unpublished studies and materials were not considered. Methodological appraisal of each study was conducted according to PRISMA standard (See File S1 for PRISMA checklist). Due to the non-experimental design and substantial variations in outcomes, such as bacterial count and DNA, across the included studies, findings will be presented by narration instead of pooled statistical meta-analysis methods.

### 2.2. Study Quality Assessment

The quality assessment of the included studies was completed using the 12 selected items from the Strengthening the Reporting of Observational Studies in Epidemiology (STROBE) guidelines [19]. Each included study can receive up to 12 marks based on the quality of its reporting methods (study design, setting, participants, variables, data sources/measurement, sample size, and statistical methods) and results about participants and relationships among the variables. Higher total scores indicate better study quality.

## 3. Results

**Figure 1:**
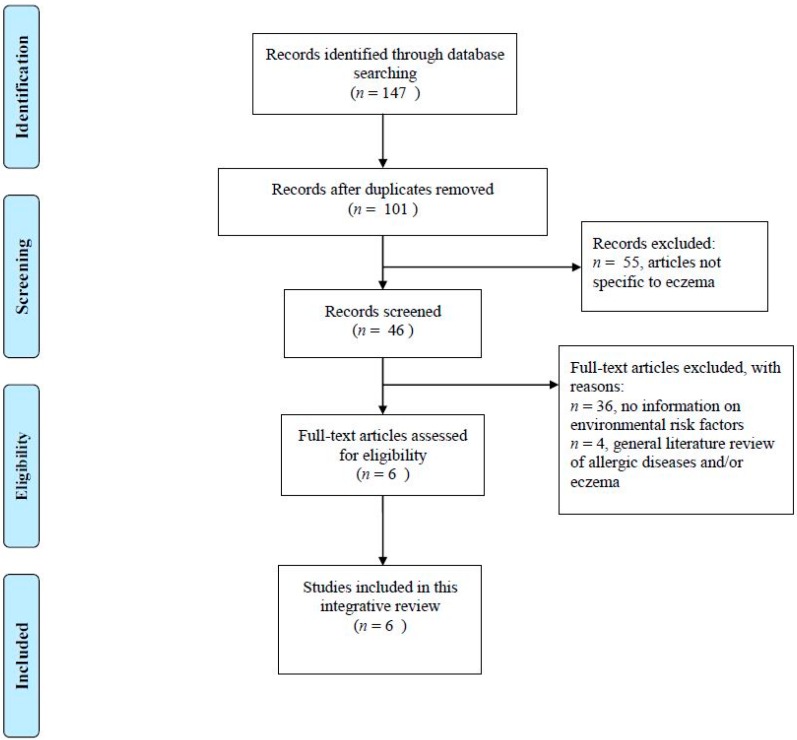
Flow diagram.

After removing duplicates, forty-six articles were retrieved from the electronic databases. Figure 1 is the flow diagram of this review. After an initial screening for suitability, six original studies were selected for review. All of them studied external environmental influences on microbial diversity in children with eczema and the role of gut microbiome in the development of eczema. The review articles (*n* = 4) and other related publications (*n* = 36) with no information on environmental risk factors were excluded. See File S2 for a list of excluded publications.

### 3.1. Study Appraisals

The six articles included in this review had a total of 1990 participants and were conducted in eight countries, including Australia, Sweden, Great Britain, Italy, Norway, Germany, and the Netherlands (Table 1). Four [20,21,22,23] were prospective cohort studies assessing the eczema status and bacterial DNA from feces of an unselected population of children. Two were case-control studies comparing the microbial composition of healthy infant participants (controls) against participants with eczema (cases). Participants received no intervention or targeted treatment. Subjects were identified as cases if eczema was present. Eczema was diagnosed by clinical professionals in all studies except one study [23] using parent report information about the occurrence of skin inflammation in order to classify child eczema status. In addition, three studies [21,24,25] also evaluated the eczema severity of the children in the study using the Scoring Atopic Dermatitis (SCORAD) scale. In terms of infant specimen samples, two studies [5,21,24,26] collected feces for bacterial DNA extraction, and four studies analyzed blood samples for quantification of atopic sensitization in addition to fecal samples [20,22,23,25]. Basic biochemical or genetic tests (subculture, restriction fragment length polymorphism, Gram staining, and fluorescent in situ hybridization) were employed to determine the microbial profiles in most studies [21,24], whereas there were a few studies employing sophisticated technology, such as quantitative real-time PCR and Sanger sequencing to determine the microbial profile [20,22,23]. However, there was only one study employed the high throughput 16S rRNA 454 pyrosequencing technology [25]. West et al. (2015) also collected stool samples from the pregnant mother at inclusion to examine the relationship between maternal and infant gut microbiome patterns [25].

Overall, the methodological quality of the included studies was acceptable, with three studies [22,23,24] scoring 11 out of a possible 12 (Table 2). All of the studies reported a clear description of study setting, participants, measurement of outcomes, and statistical analysis plan. However, none of the studies provided information on sample size estimation. Of these six individual studies, four [21,22,23,24] reported adjusted associations between microbial diversity and eczema, and two used random assignment/selection of children with eczema into control and case groups. Table 3 presents the sequence of the 16S rRNA primers and probes.

### 3.2. Environmental Factors Associated with Low Microbial Diversity Specific to Eczema

The literature included in this review highlighted the protective effect of microbiome diversity against the development of eczema. Several environmental factors have been identified to influence the composition of gut microbiota and, hence, the host’s susceptibility to infections and allergies, such as eczema. The associated environmental factors are discussed below.

#### 3.2.1. Length of Gestation

The prospective cohort study of 94 infants in Norway by Storrø et al. (2011) [20] observed alterations in gut microbiota composition and fewer premature births, although statistical significance was not specified, among infants with atopic sensitization. Reduced diversity in the early gut microbiota is associated with atopic eczema [18]. Acquisition of certain bacteria can increase tolerance against antigens, thereby reducing the infant predisposition to allergy [21].

#### 3.2.2. Mode of Delivery

Infants born through Caesarean section (CS) have been found to have a different gut microbiome composition compared with those delivered vaginally, with lower counts of *E. coli*, bifidobacteria, and *Bacteroides* species found in CS infants because of reduced contact with the maternal vaginal, perineal, and fecal flora [21,25]. The authors stated the difficulty for CS infants to acquire *E. coli* species which usually pass from mother to infant during vaginal delivery [21]. The confounding effect of delivery mode was also commonly adjusted in the analysis of the relationship between microbial diversity and eczema [21,23,24]. Penders et al. (2013) [22] demonstrated that the delivery mode influenced the development of eczema not directly but indirectly via *Clostridium* cluster I colonization. Moreover, while the bacterial communities of vaginal-delivered infants resembled their own mothers’ vaginal microbiota, CS infants had bacterial composition similar to those found on the skin surface [22].

#### 3.2.3. Type of Feeding

Infant feeding type plays an important role in the development of eczema. Breastfeeding is a well-known factor for microbial exposure in infancy with transient differences observed in colonization pattern for specific bacterial species at different ages [20]. It has been demonstrated that breastfeeding with duration of six months or longer increased the prevalence of colonization by lactobacilli and bifidobacteria [22]. Breastfeeding was considered as a potential confounding factor in several included studies [21,23,24].

#### 3.2.4. Treatment

It has been reported that children with allergies were more likely to receive antibiotic treatment [18]. This finding can be explained by the role of antibiotics in changing the host microbiota, with a modulating effect on the infant’s immune system when introduced to women in and after pregnancy or to children in defined periods early in life [20]. The other studies investigating microbial relationship with eczema also took into account the confounding effects of antibiotic [24] and probiotic use [23] during pregnancy. However, some probiotic interventions were able to modify eczema risk but not to modulate atopic sensitization, possibly because the development of infant eczema, but not atopic sensitization, can be influenced by modulation of microbiota patterns in early life [24].

#### 3.2.5. Number of Older Siblings

Another non-host factor influencing the colonization of the gut microbiota as identified from the reviewed studies is the number of older siblings. It has been found that a lack of older siblings was associated with rapid colonization by *Clostridium* species and a lower strict anaerobic/facultative anaerobic ratio at 12 months of age [21]. The colonization pattern of children without siblings was similar to that of infants born by means of CS [21,22]. A cohort study conducted in Germany found that the colonization rates of lactobacilli and bacteroides increased with the number of older siblings, indicating a dose-response relationship between birth order and microbiota composition [22]. Given the important role of gut microbiota composition in immune development, having older siblings may modulate the risk of developing eczema, and it has been considered as a potential confounding factor in our reviewed studies [24].

#### 3.2.6. Other Factors

Other non-host factors such as diet, medication, smaller family sizes, and increased hygiene may also influence the development of immunologic tolerance through changing the microbiome composition [22]. The examination of the gut microbiota composition in pregnant atopic mothers also revealed different microbial patterns in mothers whose infants had allergies compared with those whose infants remained free of allergies [25].

## 4. Discussion

We conducted this first systematic review with purposely-stringent study inclusion criteria in order to narrow down the focus and scope of our review specific to environmental influences on the composition of gut microbiome associated with eczema, which makes our review unique and different from previous reviews. With concentrated insights our review reinforces the importance of research into environmental insults that may impair immune functions through their influences on the diversity of microbiota in the host gut which in turn may increase the host risk of developing eczema. Among the reviewed studies, only Penders et al. (2013) examined the impact of non-host factors on eczema through bacterial colonization patterns [23]. Although other reviewed studies did not explore the underlying mechanisms, they acknowledged and took into consideration the confounding effects of environmental exposures that were commonly reported to have association with gut microbiome composition among children with eczema. Based upon these known risk exposures, further research is needed to elucidate how environmental exposures may alter gut microbiome patterns which, in turn, increases the host’s risk of eczema.

It should be noted that some environmental factors, such as number of older siblings can be correlated with a number of factors such as size of residential area, household income and parents’ educational level. All of these potential confounders should be considered and adjusted for in the regression analyses of the association between gut microbiota and eczema. Moreover, caution should be taken when interpreting findings on microbiota in different age groups. Environmental influences may have been stronger on the gut colonization process during infancy than later periods.

The advancement of high throughput sequencing allows for high-speed survey and quantification of the functions of microbes. With the advances in Next Generation Sequencer such as 454 FLX, IonTorrent PGM, MiSeq, and NextSeq500, high throughput 16S ribosomal sequencing is now gaining popularity and becoming a fundamental approach for the microbial diversity analysis. However, it should be noted that different reports of eczema-related microbiota profile could be due to different research approaches employed in the studies. Most of the microbial commensals cannot be cultured and the numerous sequencing reads produced in a single 16S experiment are able to generate a microbial profile down to genera, or even species, level. For example, reduced diversity of gut commensals was observed only in the study of West et al. (2015) [25] which was based on a high-throughput metagenomics approach. The primers used in the high throughput metagenomics approach are universal across most gut commensals and the data generated are in terms of a few thousand sequencing reads representing the diversity of the whole gut microbiota. However, the studies of Pender et al. (2013) [22] and Storrø, et al. (2011) [20] focused only on a selected group of representative target bacteria, but not on the whole gut symbiotic community.

With the new imaging tools, researchers can devote more resources and time to the research of the human microbiome. Previous studies provided some evidence for the differences in the composition of gut microbiota between children with and without eczema [25,26]. The gut microbiota is important for human health because of its role in shaping the immune system early in life [15]. It has been suggested that in addition to the composition, the site on which the bacteria is sampled also influences the manifestation of the inflammation in certain skin areas [17]. On the other hand, the colonization of the microbiota in the gut is subject to the influences in the host environment [16]. The resulting alterations of the colonization process can predispose the host to certain infections and allergies because of impaired immune development. However, the mechanism linking environmental influences, microbial composition, and eczema remains inconclusive. The gut microbiome may act as a mediator influencing the relationship between eczema risk and individual risk factors such as delivery mode and birth order [22]. There is also conflicting evidence as to the modulatory effects of gut microbiota on immunological functions. Ismail et al. (2012) [24] found that low microbial diversity increased eczema risk regardless of the infant atopic sensitization status, whereas Storrø et al. (2011) [20] observed age-dependent microbiome influences on atopic sensitization but not atopic eczema.

As the environmental determinants of eczema remain inconclusive, and gut commensals are essential for healthy immune development, with gut microbiota dysbiosis often linked with the etiology of the disease, further large-scaled prospective birth cohort studies are needed to explore the gut microbiome development using the metagenomic approach and to look into the direct effect of environmental factors, such as mode of delivery, type of feeding, sibling size, early exposure to antibiotics, and type of pets on the development of eczema. Moreover, the question of how maternal variables such as maternal age and medications, as well as immunoglobulins and other components of breast milk, would affect the infant’s gut microbiota and eczema risk remains largely unclear. Answers to these questions would be beneficial for more in-depth understanding of the disease pathology.

### 4.1. Strength and Limitations

This is the first systematic and critical review of the primary studies pertaining to the role of gut microbiota in the development of eczema and how its composition is influenced by environmental and individual risk factors. The included articles were systematically scored and critically appraised to reflect the methodological quality and scientific value of the study findings.

Although the included studies provided clear descriptive background and concepts of gut microbiota in association with eczema, we cannot draw conclusions about the strength of the association because the heterogeneity in design and quality of the selected studies makes it impossible to formally calculate pooled estimates of the associations by using the meta-analysis methods. For instance, most studies were primarily designed to study the microbial composition differences between healthy and eczema subjects with statistical adjustment for the environmental and individual factors. Although they addressed the potential confounding effects from the environment, their primary study goal was not to investigate the direct influences of these environmental variables on microbe-induced immune responses and, therefore, we cannot ascertain the cause and effect relationships among study variables.

### 4.2. Implication for Future Research and Practice

Eczema is often the first and main sign of atopic disease in the first two years of life [27]. Further large scale, longitudinal, and multifaceted empirical studies are needed to investigate the relationship between environmental exposures, microbiome composition, and eczema using the metagenomic approach. The results generated from these studies will provide greater insights into the epidemiology of eczema and help healthcare professionals to develop new strategies to prevent and manage eczema. Moreover, most current findings and literature pertains to the situation in the developed countries. Examinations of the environmental-host-microbial interaction and its role in health and disease in other cultures and regions are critical for establishing a comprehensive knowledge base for mitigating the growth of allergic diseases around the world.

## 5. Conclusions

As the immune system of infants is not fully functional at birth, they are susceptible to invading microbes from the environment. Existing evidence suggests that the gut microbiota plays an important role in protecting the host from infections and allergies. It is important to detect the gene-environmental interactions, as well as the environmental risk factors contributing to low microbial diversity in early childhood, which might predispose the child to eczema.

## Figures and Tables

**Table 1:** Characteristics of original studies.

Study	Study Type	Study Place	Participants	Data Collection Time		Outcome Measures	Techniques Used to Determine the Microbial Diversity	Results
				Evaluation of Eczema	Collection of Samples			
Penders et al. (2007) [23]	Prospective cohort study	The Netherlands	957 infants	Postpartum questionnaires on child eczema status in the seven, 12 and 24 months	Infant feces collected at age one month; Infant blood samples collected at age two years	Bacteria counts, total and specific IgE, atopic manifestations and sensitization	Quantitative real-time PCR	(1) More *Escherichia coli* was associated with higher eczema risk after adjusting for subcohort, parental history of atopy, sibling history of atopy, age at collection of fecal sample, infant’s gender and total bacterial count; (2) infants colonized with *Clostridium difficile* were at higher risk of developing eczema, recurrent wheeze and allergic sensitization; and (3) the presence of *C difficile* was associated with higher eczema risk
Adlerberth et al. (2007) [21]	Prospective cohort study	Multiple	116 Goteborg children, 108 London children, 100 Rome children	Atopic eczema and total and food-specific IgE levels assessed at age 18 months	Rectal swab collected at age three days; Fecal samples collected at seven, 14, and 28 days and age two, six, and 12 months	Atopic eczema diagnosis according to Williams’ criteria, SCORAD, Serum total and specific IgE levels against common food antigens	Subculture, Gram staining and biochemical/genetic tests	(1) No association between atopic eczema or food-specific IgE by age 18 months and any particular bacterial group acquisition time after adjusting for mode of delivery, parity, and breastfeeding at six months; (2) Cesarean section delayed colonization by *Escherichia coli* and *Bacteroides* and *Bifidobacterium* species, giving way to *Clostridium*; and (3) Lack of older siblings was associated with earlier colonization by *Clostridium* species and lower strict anaerobic/facultative anaerobic ratio at 12 months after adjusting for mode of delivery, parity, breastfeeding at six months, and dietary correlates.
Storrø et al. (2011) [20]	Prospective cohort study	Norway	94 infants	Eczema survey assessment at six weeks after delivery and at age one and two years	Infant venous blood for slgE quantification collected at age two years; Infant feces collected at 10 days postnatal, age 4 months and 1 and 2 years	Eczema diagnosis using UK Working Party (UKWP) criteria, slgE concentrations, microbiome composition analyzed through feces samples	Quantitative real-time PCR	(1) Less *Escherichia coli* at four months and one year and Bacteroides fragilis at two years plus more *Bifidobacterium longum* at one year among subjects with atopic sensitization; and (2) Higher likelihood of family history of atopy, antibiotic treatment and breastfeeding plus lower likelihood of parental smoking among subjects with atopic sensitization; (3) no association between colonization patterns and atopic eczema.
Ismail et al. (2012) [24]	Case-control study	Australia	cases: 33 (33.7%) infants with eczema; controls: 65 (66.3%) healthy infants	Infants evaluated at three, six, and 12 months for the presence of eczema	Infant fecal samples collected at day three, seven, 28, 90 and 180 of life	Skin prick testing, the Scoring Atopic Dermatitis (SCORAD) scale, bacterial DNA from infant fecal specimens	Terminal restriction fragment length polymorphism (T-RFLP)	(1) At day seven, higher microbial diversity in healthy infants than infants with eczema at age 12 months after adjusting for mode of delivery, number of siblings, antibiotics use during pregnancy, breastfeeding, household pets, and maternal allergy
Penders et al. (2013) [22]	Prospective cohort study	Germany	497 infants	Infants evaluated regularly by a pediatrician for atopic dermatitis (AD) signs from the start of intervention until age three years	Fecal samples collected at age five weeks, 13 weeks and 31 weeks	Bacterial DNA from faeces, AD diagnosis	Quantitative real-time PCR	(1) Higher colonization rates of lactobacilli and bacteroides but lower rates of clostridia at age five weeks were associated with more older siblings; (2) colonization of clostridia at age five and 13 weeks was associated with higher eczema risk in the subsequent six months of life; and (3) indirect effect of *Clostridium* cluster I colonization on birth mode and birth order in association to eczema
West et al. (2015) [25]	Case-control study	Australia	cases: 10 children with IgE-associated eczema; controls: 10 non-allegic children	Infants evaluated at age six and 12 months and 2.5 years	Stool samples collected from pregnant mother at inclusion and infant at age one week, one month, and 12 months	Skin prick testing, the Scoring Atopic Dermatitis (SCORAD) scale, bacterial DNA from infant fecal specimens	16S rRNA 454 pyrosequencing	(1) Association between reduced relative abundance of potentially immunomodulatory gut bacteria and exaggerated inflammatory cytokine responses to TLR-ligands and subsequent development of IgE-associated eczema; (2) More proteobacteria and less bacteroidetes in caesarean section born children than vaginally delivered children; and (3) More bacteroidetes in stool samples of mothers whose infants developed IgE-associated eczema and also in infants later diagnosed with atopic eczema of previous study

**Table 2:** Quality assessment of original studies. * means the study received 1 mark for that selected item.

Study	Methods								Results				Total Quality Score
	Study Design (Report Key Elements?)	Setting (Report Study Setting Details?)	Participants (Give Eligibility Criteria?)	Variables (Define All Variables?)	Data Sources/Measurement (Give Sources of Data?)	Bias (Address Potential Bias?)	Sample Size (How to Measure Study Size?)	Statistical Methods (Describe Statistical Methods Used?)	Participants (Report the Numbers of Individuals at Each Study Stage?)	Descriptive Data (Report Study Subject Characteristics?)	Outcome Data (Report Number in Each Exposure Category?)	Main Results (Report Unadjusted Estimates?)	
Penders et al. (2007) [23]	*	*	*	*	*	*	–	*	*	*	*	*	11
Adlerberth et al. (2007) [21]	*	*	*	*	*	–	–	*	*	*	*	*	10
Storrø et al. (2011) [20]	*	*	*	*	*	–	–	*	*	*	*	–	9
Ismail et al. (2012) [24]	*	*	*	*	*	*	–	*	*	*	*	*	11
Penders et al. (2013) [22]	*	*	*	*	*	*	–	*	*	*	*	*	11
West et al. (2015) [25]	*	*	*	*	*	*	–	*	*	*	*	–	10

**Table 3:** Sequence of 16S rRNA primers and probes.

Study	Target Organisms	Primer/Probe	Sequence (5′-3′)
Penders et al. (2007) [23]	*Bifidobacterium* spp.	Forward primer	GCGTGCTTAACACATGCAAGTC
		Reverse primer	CACCCGTTTCCAGGAGCTATT
		Probe	TCACGCATTACTCACCCGTTCGCC
	*Escherichia coli*	Forward primer	CATGCCGCGTGTATGAAGAA
		Reverse primer	CGGGTAACGTCAATGAGCAAA
		Probe	TATTAACTTTACTCCCTTCCTCCCCGCTGAA
	*Clostridium difficile*	Forward primer	TTGAGCGATTTACTTCGGTAAAGA
		Reverse primer	TGTACTGGCTCACCTTTGATATTCA
		Probe	CCACGCGTTACTCACCCGTCCG
	*Bacteroides fragilis* group	Forward primer	CGGAGGATCCGAGCGTTA
		Reverse primer	CCGCAAACTTTCACAACTGACTTA
		Probe	CGCTCCCTTTAAACCCAATAAATCCGG
	*Lactobacillus* spp.	Forward primer	AGCAGTAGGGAATCTTCCA
		Reverse primer	CACCGCTACACATGGAG
Storrø, et al. (2011b) [20]	Bacteria	Forward primer	TCCTACGGGAGGCAGCAGT
		Probe	FAM-CTGATTACCGCGGCTGCTGGCAC-TAMRA
		Reverse primer	GGACTACCAGGGTATCTAATCCTGTT
	*Bacteroides fragilis*	Forward primer	GAAAGCATTAAGTATTCCACCTG
		Probe	FAM-TGAAACTCAAAGGAATTGACGGGG-TAMRA
		Reverse primer	CGGTGATTGGTCACTGACA
	*Escherichia coli*	Forward primer	GTGTGATATCTACCCGCTTCGC
		Probe	FAM-TCGGCATCCGGTCAGTGGCAGT-TAMRA
		Reverse primer	AGAACGGTTTGTGGTTAATCAGGA
	*Bifidobacterium breve*	Forward primer	GTGGTGGCTTGAGAACTGGATAG
		Probe	FAM-TGATTCCTCGTTCTTGCTGT-MGB
		Reverse primer	CAAAACGATCGAAACAAACACTAAA
	*Bifidobacterium lactis*	Forward primer	AGAACCACGGCGGCGTC
		Probe	FAM-TGCGCTCGCCGACG-MGB
		Reverse primer	CGCGGTCTTCTCGAGCACT
	*Bifidobacterium longum*	Forward primer	TGGAAGACGTCGTTGGCTTT
		Probe	FAM-CGCACCCACCGCA-MGB
		Reverse primer	ATCGCGCCAGGCAAAA
	*Bifidobacterium* spp.	Forward primer	GGGATGCTGGTGTGGAAGAGA
		Probe	FAM-TCAAACCACCACGCGCCA-MGB
		Reverse primer	TGCTCGCGTCCACTATCCAGT
	*Lactobacillus reuteri*	Forward primer	ATGGCTTTTGTTTGAAAGATGGC
		Probe	FAM-TGGCTATCACTCTGGGATG-MGB
		Reverse primer	CCTTACCAACTAGCTAATGCACCG
	*Lactobacillus rhamnosus*	Forward primer	CATAAATCCAAGAACCGCATGG
		Probe	FAM-CTTGGCTGAAAGATG-MGB
		Reverse primer	CACGCCGACAACAGTTACTCTGC
	*Clostridium difficile*	Forward primer	ATATCAGAGACTGATGAG
		Probe	FAM-TGGAGAATCTATATTTGTAGAAAC-MGB
		Reverse primer	TAGCATATTCAGAGAATATTGT
	*Clostridium perfringens*	Forward primer	TTCTATCTTGGAGAGGCTATGCACTATTTT
		Probe	FAM-TAGATACTCCATATCATCCTGCTAATGTTACTGCCGTTGA-TAMRA
		Reverse primer	TTTCAAACTTAACATGTCCTGCGC
	*Lactobacillus plantarum*	Forward primer	TGGACCGCATGGTCCGAG
		Probe	FAM-TCCCGCGGCGTATTA-MGB
		Reverse primer	GTGAGCCGTTACCCCACCAT
	*Helicobacter pylori*	Forward primer	CGTGGCAAGCATGATCCAT
		Probe	FAM-TCAGGAAACATCGCTTCAATACCCACTT-TAMRA
		Reverse primer	GGGTATGCACGGTTACGAGTTT
Penders et al. (2013) [22]	*Bifidobacterium* spp.	Forward primer	GCGTGCTTAACACATGCAAGTC
		Reverse primer	CACCCGTTTCCAGGAGCTATT
		Probe	TCACGCATTACTCACCCGTTCGCC
	*E. coli*	Forward primer	CATGCCGCGTGTATGAAGAA
		Reverse primer	CGGGTAACGTCAATGAGCAAA
		Probe	TATTAACTTTACTCCCTTCCTCCCCGCTGAA
	*C difficile*	Forward primer	TTGAGCGATTTACTTCGGTAAAGA
		Reverse primer	TGTACTGGCTCACCTTTGATATTCA
		Probe	CCACGCGTTACTCACCCGTCCG
	*B fragilis* group	Forward primer	CGGAGGATCCGAGCGTTA
		Reverse primer	CCGCAAACTTTCACAACTGACTTA
		Probe	CGCTCCCTTTAAACCCAATAAATCCGG
	*Lactobacillus* spp.	Forward primer	AGCAGTAGGGAATCTTCCA
		Reverse primer	CACCGCTACACATGGAG
	*Clostridium* cluster I	Forward primer	TACCHRAGGAGGAAGCCAC
		Reverse primer	GTTCTTCCTAATCTCTACGCAT
		Probe	GTGCCAGCAGCCGCGGTAATACG

## Supplementary Materials

Supplementary materials can be found at http://www.mdpi.com/1422-0067/17/7/1147/s1.
