# Supplementary material for: Environmental Factors Associated with Altered Gut Microbiota in Children with Eczema: A Systematic Review
Source: Int J Mol Sci. 2016 Jul 16;17(7):1147. doi: 10.3390/ijms17071147 (PMC4964520; doi:10.3390/ijms17071147)
Supplement: Supplementary file 1 [file ijms-17-01147-s001.pdf]

Supplementary Materials: Environmental Factors Associated with Altered Gut Microbiota in Children with Eczema: A Systematic Review

Carmen W.H. Chan, Rosa S. Wong, Patrick T.W. Law, Cho Lee Wong, Stephen K.W. Tsui, Winnie P.Y. Tang and Janet W.H. Sit

File S1. PRISMA checklist [1].

Section/Topic	Item Number	Checklist Item	Reported on Page Number
**Title**			
Title	1	Identify the report as a systematic review, meta-analysis, or both.	1
**Abstract**			
Structured summary	2	Provide a structured summary including, as applicable: background; objectives; data sources; study eligibility criteria, participants, and interventions; study appraisal and synthesis methods; results; limitations; conclusions and implications of key findings; systematic review registration number.	1
**Introduction**			
Rationale	3	Describe the rationale for the review in the context of what is already known.	1–2
Objectives	4	Provide an explicit statement of questions being addressed with reference to participants, interventions, comparisons, outcomes, and study design (PICOS).	2
**Methods**			
Protocol and registration	5	Indicate if a review protocol exists, if and where it can be accessed (e.g., Web address), and, if available, provide registration information including registration number.	2–3
Eligibility criteria	6	Specify study characteristics (e.g., PICOS, length of follow-up) and report characteristics (e.g., years considered, language, publication status) used as criteria for eligibility, giving rationale.	2–3
Information sources	7	Describe all information sources (e.g., databases with dates of coverage, contact with study authors to identify additional studies) in the search and date last searched.	2
Search	8	Present full electronic search strategy for at least one database, including any limits used, such that it could be repeated.	2–3

File S1. *Cont.*

Section/Topic	Item Number	Checklist Item	Reported on Page Number
Study selection	9	State the process for selecting studies (i.e., screening, eligibility, included in systematic review, and, if applicable, included in the meta-analysis).	2–3
Data collection process	10	Describe method of data extraction from reports (e.g., piloted forms, independently, in duplicate) and any processes for obtaining and confirming data from investigators.	2–3
Data items	11	List and define all variables for which data were sought (e.g., PICOS, funding sources) and any assumptions and simplifications made.	2–3
Risk of bias in individual studies	12	Describe methods used for assessing risk of bias of individual studies (including specification of whether this was done at the study or outcome level), and how this information is to be used in any data synthesis.	3
Summary measures	13	State the principal summary measures (e.g., risk ratio, difference in means).	NA Nil meta-analysis
Synthesis of results	14	Describe the methods of handling data and combining results of studies, if done, including measures of consistency (e.g., I^2) for each meta-analysis.	NA Nil meta-analysis
Risk of bias across studies	15	Specify any assessment of risk of bias that may affect the cumulative evidence (e.g., publication bias, selective reporting within studies).	3
Additional analyses	16	Describe methods of additional analyses (e.g., sensitivity or subgroup analyses, meta-regression), if done, indicating which were pre-specified.	NA Nil meta-analysis
**Results**			
Study selection	17	Give numbers of studies screened, assessed for eligibility, and included in the review, with reasons for exclusions at each stage, ideally with a flow diagram.	3
Study characteristics	18	For each study, present characteristics for which data were extracted (e.g., study size, PICOS, follow-up period) and provide the citations.	5–6
Risk of bias within studies	19	Present data on risk of bias of each study and, if available, any outcome level assessment (see Item 12).	7
Results of individual studies	20	For all outcomes considered (benefits or harms), present, for each study: (a) simple summary data for each intervention group; (b) effect estimates and confidence intervals, ideally with a forest plot.	NA Nil meta-analysis

File S1. *Cont.*

Section/Topic	Item Number	Checklist Item	Reported on Page Number
Synthesis of results	21	Present results of each meta-analysis done, including confidence intervals and measures of consistency.	NA Nil meta-analysis
Risk of bias across studies	22	Present results of any assessment of risk of bias across studies (see Item 15).	7
Additional analysis	23	Give results of additional analyses, if done (e.g., sensitivity or subgroup analyses, meta-regression (see Item 16)).	NA Nil meta-analysis
**Discussion**			
Summary of evidence	24	Summarize the main findings including the strength of evidence for each main outcome; consider their relevance to key groups (e.g., healthcare providers, users, and policy makers).	11–12
Limitations	25	Discuss limitations at study and outcome level (e.g., risk of bias), and at review-level (e.g., incomplete retrieval of identified research, reporting bias).	12
Conclusions	26	Provide a general interpretation of the results in the context of other evidence, and implications for future research.	12
**Funding**			
Funding	27	Describe sources of funding for the systematic review and other support (e.g., supply of data); role of funders for the systematic review.	NA Nil funding

[1] From reference [28]. For more information, available online: www.prisma-statement.org.

File S2. List of excluded publications.

1	Abrahamsson, T.R.; Jakobsson, H.E.; Andersson, A.F.; Bjorksten, B.; Engstrand, L.; Jenmalm, M.C. Low diversity of the gut microbiota in infants with atopic eczema. *J. Allergy Clin. Immunol.* **2012**, *129*, 434–440.
2	Abrahamsson, T.R.; Jakobsson, H.E.; Andersson, A.F.; Bjorksten, B.; Engstrand, L.; Jenmalm, M.C. Low gut microbiota diversity in early infancy precedes asthma at school age. *Clin. Exp. Allergy* **2014**, *44*, 842–850.
3	Arvola, T.; Ruuska, T.; Keranen, J.; Hyoty, H.; Salminen, S.; Isolauri, E. Rectal bleeding in infancy: Clinical, allergological, and microbiological examination. *Pediatrics* **2006**, *117*, e760–e768.
4	Ashley, S.; Dang, T.; Koplin, J.; Martino, D.; Prescott, S. Food for thought: Progress in understanding the causes and mechanisms of food allergy. *Curr. Opin. Allergy Clin. Immunol.* **2015**, *15*, 237–242.
5	Azad, M.B.; Becker, A.B.; Guttman, D.S.; Sears, M.R.; Scott, J.A.; Kozyrskyj, A.L. Gut microbiota diversity and atopic disease: Does breast-feeding play a role? *J. Allergy Clin. Immunol.* **2013**, *131*, 247–248.
6	Bendiks, M.; Kopp, M.V. The relationship between advances in understanding the microbiome and the maturing hygiene hypothesis. *Curr. Allergy Asthma Rep.* **2013**, *13*, 487–494.
7	Bjorksten, B. Disease outcomes as a consequence of environmental influences on the development of the immune system. *Curr. Opin. Allergy Clin. Immunol.* **2009**, *9*, 185–189.
8	Cosenza, L.; Nocerino, R.; Di Scala, C.; Di Costanzo, M.; Amoroso, A.; Leone, L.; Paparo, L.; Pezzella, C.; Aitoro, R.; Berni Canani, R. Bugs for atopy: The Lactobacillus rhamnosus GG strategy for food allergy prevention and treatment in children. *Benef. Microbes* **2015**, *6*, 225–232.
9	Forno, E.; Onderdonk, A.B.; McCracken, J.; Litonjua, A.A.; Laskey, D.; Delaney, M.L.; DuBois, A.M.; Gold, D.R.; Ryan, L.M.; Weiss, S.T.; et al. Diversity of the gut microbiota and eczema in early life. *Clin. Mol. Allergy* **2008**, *6*, doi:10.1186/1476-7961-6-11.
10	Gosalbes, M.J.; Llop, S.; Valles, Y.; Moya, A.; Ballester, F.; Francino, M.P. Meconium microbiota types dominated by lactic acid or enteric bacteria are differentially associated with maternal eczema and respiratory problems in infants. *Clin. Exp. Allergy* **2013**, *43*, 198–211.
11	Ismail, I.H.; Oppedisano, F.; Joseph, S.J.; Boyle, R.J.; Robins-Browne, R.M.; Tang, M.L. Prenatal administration of Lactobacillus rhamnosus has no effect on the diversity of the early infant gut microbiota. *Pediatr. Allergy immunol.* **2012**, *23*, 255–258.
12	Isolauri, E.; Ouwehand, A.C.; Laitinen, K. Novel approaches to the nutritional management of the allergic infant. *Acta Paediatr.* **2005**, *94*, 110–114.
13	Isolauri, E.; Rautava, S.; Salminen, S. Probiotics in the development and treatment of allergic disease. *Gastroenterol. Clin. N. Am.* **2012**, *41*, 747–762.
14	Isolauri, E.; Salminen, S. The impact of early gut microbiota modulation on the risk of child disease: Alert to accuracy in probiotic studies. *Benef. Microbes* **2015**, *6*, 167–171.
15	Kalliomaki, M.; Antoine, J.M.; Herz, U.; Rijkers, G.T.; Wells, J.M.; Mercenier, A. Guidance for substantiating the evidence for beneficial effects of probiotics: Prevention and management of allergic diseases by probiotics. *J. Nutr.* **2010**, *140*, 713s–721s.
16	Kalliomaki, M.; Isolauri, E. Role of intestinal flora in the development of allergy. *Curr. Opin. Allergy Clin. Immunol.* **2003**, *3*, 15–20.
17	Kalliomaki, M.; Salminen, S.; Isolauri, E. Positive interactions with the microbiota: Probiotics. *Adv. Exp. Med. Biol.* **2008**, *635*, 57–66.
18	Kirjavainen, P.V.; Arvola, T.; Salminen, S.J.; Isolauri, E. Aberrant composition of gut microbiota of allergic infants: A target of bifidobacterial therapy at weaning? *Gut* **2002**, *51*, 51–55.
19	Kirjavainen, P.V.; Salminen, S.J.; Isolauri, E. Probiotic bacteria in the management of atopic disease: Underscoring the importance of viability. *J. Pediatr. Gastroenterol. Nutr.* **2003**, *36*, 223–227.

File S2. *Cont.*

20	Kukkonen, K.; Savilahti, E.; Haahtela, T.; Juntunen-Backman, K.; Korpela, R.; Poussa, T.; Tuure, T.; Kuitunen, M. Probiotics and prebiotic galacto-oligosaccharides in the prevention of allergic diseases: A randomized, double-blind, placebo-controlled trial. *J. Allergy Clin. Immunol.* **2007**, *119*, 192–198.
21	Laiho, K.; Ouwehand, A.; Salminen, S.; Isolauri, E. Inventing probiotic functional foods for patients with allergic disease. *Ann. Allergy Asthma Immunol.* **2002**, *89*, 75–82.
22	Laursen, M.F.; Zachariassen, G.; Bahl, M.I.; Bergström, A.; Høst, A.; Michaelsen, K.F.; Licht, T.R. Having older siblings is associated with gut microbiota development during early childhood. *BMC Microbiol.* **2015**, *15*, 154.
23	Munyaka, P.M.; Khafipour, E.; Ghia, J.E. External influence of early childhood establishment of gut microbiota and subsequent health implications. *Front. Pediatr.* **2014**, *2*, 109.
24	Nermes, M.; Salminen, S.; Isolauri, E. Is there a role for probiotics in the prevention or treatment of food allergy? *Curr. Allergy Asthma Rep.* **2013**, *13*, 622–630.
25	Penders, J.; Stobberingh, E.E.; Thijs, C.; Adams, H.; Vink, C.; Van Ree, R.; Van Den Brandt, P.A. Molecular fingerprinting of the intestinal microbiota of infants in whom atopic eczema was or was not developing. *Clin. Exp. Allergy* **2006**, *36*, 1602–1608.
26	Penders, J.; Stobberingh, E.E.; van den Brandt, P.A.; Thijs, C. The role of the intestinal microbiota in the development of atopic disorders. *Allergy* **2007**, *62*, 1223–1236.
27	Penders, J.; Thijs, C.; Mommers, M.; Stobberingh, E.E.; Dompeling, E.; Reijmerink, N.E.; van den Brandt, P.A.; Kerkhof, M.; Koppelman, G.H.; Postma, D.S. Host-microbial interactions in childhood atopy: Toll-like receptor 4 (TLR4), CD14, and fecal *Escherichia coli*. *J. Allergy Clin. Immunol.* **2010**, *125*, 231–236.
28	Prince, B.T.; Mandel, M.J.; Nadeau, K.; Singh, A.M. Gut microbiome and the development of food allergy and allergic disease. *Pediatr. Clin. N. Am.* **2015**, *62*, 1479–1492.
29	Roberfroid, M.; Gibson, G.R.; Hoyles, L.; McCartney, A.L.; Rastall, R.; Rowland, I.; Wolvers, D.; Watzl, B.; Szajewska, H.; Stahl, B.; Guarner, F. Prebiotic effects: Metabolic and health benefits. *Br. J. Nutr.* **2010**, *104* (Suppl. S2), S1–S63.
30	Singh, M.; Ranjan Das, R. Probiotics for allergic respiratory diseases—Putting it into perspective. *Pediatr. Allergy Immunol.* **2010**, *21*, e368–e376.
31	Szajewska, H. Early nutritional strategies for preventing allergic disease. *Isr. Med. Assoc. J.* **2012**, *14*, 58–62.
32	Tang, M.F.; Sy, H.Y.; Kwok, J.S.L.; Tam, W.H.; Hon, K.L.; Tung, C.K.C.; Wong, G.W.K.; Tsui, S.K.W.; Leung, T.F. Eczema susceptibility and composition of faecal microbiota at four weeks of age: A pilot study in Chinese infants. *Br. J. Dermatol.* **2016**, *174*, 898–900.
33	Vael, C.; Vanheirstraeten, L.; Desager, K.N.; Goossens, H. Denaturing gradient gel electrophoresis of neonatal intestinal microbiota in relation to the development of asthma. *BMC Microbiol*. **2011**, *11*, 68.
34	Wall, R.; Ross, R.P.; Ryan, C.A.; Hussey, S.; Murphy, B.; Fitzgerald, G.F.; Stanton, C. Role of gut microbiota in early infant development. *Clin. Med. Pediatr.* **2009**, *3*, 45–54.
35	Wegienka, G.; Zoratti, E.; Johnson, C.C. The role of the early-life environment in the development of allergic disease. *Immunol. Allergy Clin. N. Am.* **2015**, *35*, 1–17.
36	Martin, R.; Nauta, A.; Ben Amor, K.; Knippels, L.; Knol, J.; Garssen, J. Early life: Gut microbiota and immune development in infancy. *Benef. Microbes* **2010**, *1*, 367–382.
37	West, C.E. Gut microbiota and allergic disease: New findings. *Curr. Opin. Clin. Nutr. Metab. Care* **2014**, 17, 261–266.
38	Marsland, B.J.; Salami, O. Microbiome influences on allergy in mice and humans. *Curr. Opin. Immunol.* **2015**, *36*, 94–100.
39	Flohr, C.; Mann, J. New insights into the epidemiology of childhood atopic dermatitis. *Allergy* **2014**, *69*, 3–16.
40	Mah, K.W.; Björkstén, B.; Lee, B.W.; Van Bever, H.P.; Shek, L.P.; Tan, T.N.; Lee, Y.K.; Chua, K.Y. Distinct pattern of commensal gut microbiota in toddlers with eczema. *Int. Arch. Allergy Immunol.* **2006**, *140*, 157–163.
